# A novel method based on ultrasound radiomics for predicting attainment of near-adult height in adolescents

**DOI:** 10.3389/fendo.2026.1925196

**Published:** 2026-09-18

**Authors:** Yixuan Zhang, Zeqing Zhao, Yuxin Sun, Li Zhang, Yamei Yang, Hui Pan, Shi Chen, Jianchu Li, Xiao Yang

**Affiliations:** 1 Department of Ultrasound, State Key Laboratory of Complex Severe and Rare Diseases, Peking Union Medical College Hospital, Chinese Academy of Medical Sciences & Peking Union Medical College, Beijing, China; 2 Department of Anesthesiology, National Cancer Center/National Clinical Research Center for Cancer/Cancer Hospital, Chinese Academy of Medical Sciences & Peking Union Medical College, Beijing, China; 3 Key Laboratory of Endocrinology of National Health Commission, Department of Endocrinology, Peking Union Medical College Hospital, Chinese Academy of Medical Sciences & Peking Union Medical College, Beijing, China; 4 Zhangzhou Municipal Hospital Affiliated to Fujian Medical University, Zhangzhou, Fujian, China

**Keywords:** adolescent growth assessment, machine learning, near-adult height, predictive model, ultrasound radiomics

## Abstract

**Objective:**

This study aimed to develop and validate a novel ultrasound radiomics model for predicting the attainment of near-adult height (NAH) in adolescents.

**Methods:**

This dual-center prospective cohort study enrolled 192 adolescents aged 11.0–18.0 years. Standardized ultrasound examinations of the ulnar growth plate region were performed, and 475 radiomic features were extracted. Feature selection was conducted using Least Absolute Shrinkage and Selection Operator (LASSO) algorithm with 5-fold cross-validation. Seven machine learning algorithms were compared to develop predictive models. Three models were constructed: clinical (sex and bone age), radiomics (Rad-score), and combined (sex, bone age, and Rad-score). Model performance was evaluated using area under the receiver operating characteristic curve, accuracy, sensitivity, specificity, calibration curves, and decision curve analysis.

**Results:**

Three stable radiomic features were selected with selection frequency ≥80%: wavelet-LH_glcm_Imc2, wavelet-LL_ngtdm_Busyness, and wavelet-HL_firstorder_Energy. The combined model demonstrated superior performance, achieving AUCs of 0.981 (95% CI: 0.948–1.000) in the validation set, with sensitivity of 90% and specificity of 100%. Multivariate analysis identified independent positive predictors including Rad-score (OR = 3.28, P = 0.013) and bone age (OR = 2.65, P < 0.001). And male sex was a significant negative predictor (OR = 0.15, P = 0.005).

**Conclusion:**

The combined ultrasound radiomics machine learning model achieved excellent predictive performance for NAH attainment in adolescents. The non-ionizing ultrasound approach enables repeated dynamic monitoring, and the derived nomogram facilitates individualized risk stratification in routine pediatric practice.

## Introduction

Adolescence is a critical stage of skeletal development in which longitudinal bone growth accelerates and the physis gradually mature and close (1, 2). During this period, clinicians are often faced with the practical challenge of accurately judging a child’s remaining growth potential, because the answer directly affects whether the child is likely to reach an ideal adult height and whether timely intervention is needed. In routine practice, growth-related disorders such as short stature and pubertal timing abnormalities remain common concerns in pediatric and adolescent health care (3–5). Therefore, a reliable method for evaluating residual growth potential is essential for individualized counseling, follow-up planning, and treatment decision-making.

Current assessment of residual growth potential still relies mainly on hand-wrist bone age (BA) radiography (2). However, the atlas-based method is not ideal for repeated large-scale screening in healthy middle school students because X-ray interpretation is subjective and prone to manual error. Although epiphyseal MRI avoids radiation, it is expensive, requires fixed equipment, and is unsuitable for adolescents with metal implants or for those who cannot tolerate the examination because of claustrophobia or Attention deficit hyperactivity disorder (ADHD) (6). In contrast, ultrasound is radiation-free, portable, and rapid, enabling bedside evaluation or on-site school health screenings (7). These advantages make it well suited for large-scale adolescent screening and repeated follow-up without safety concerns. Nevertheless, conventional two-dimensional ultrasound for epiphyseal assessment mainly provides linear measurements and lacks quantitative microstructural information, which limits its ability to predict growth potential (8). Therefore, a practical and accurate method is still needed for large-scale school-based screening.

Radiomics provides a potential solution by converting medical images into a large number of quantitative features that describe intensity, texture, and higher-order heterogeneity (9, 10). These features can reveal information that is not apparent to the naked eye and may better capture the biological status of the growth plate region. In skeletal imaging, radiomics has shown promise in extracting maturity-related signals from imaging data and supporting predictive modeling (6). Nevertheless, most previous studies have focused on hospital-based cohorts or on BA estimation rather than on clinically relevant prediction of residual growth potential in general adolescent populations (11). To address this gap, the present study was designed to develop a novel ultrasound radiomics model for evaluating growth potential and predicting whether adolescents can reach their expected adult height.

## Methods

### Study design and participant inclusion

This was a dual-center prospective observational cohort study that enrolled 200 adolescents from two secondary schools in an eastern Chinese city. Inclusion criteria were chronological age (CA) 11–18 years at the time of examination. Exclusion criteria were as follows: (1) a recent hand or wrist fracture, or joint deformity that impaired ultrasound image quality; (2) known systemic, endocrine, or developmental disorders, such as growth hormone deficiency, nutritional disorders, or chromosomal abnormalities; and (3) a history of growth hormone treatment.

Based on the inclusion and exclusion criteria, 192 participants were screened for analysis. The recruitment flowchart is shown in **Figure 1**. The study protocol was reviewed and approved by the Ethics Committee of Peking Union Medical College Hospital. The official ethics approval number is I-23PJ1239, and the hospital’s internal project acceptance number is K23C2396.

**Figure 1:**
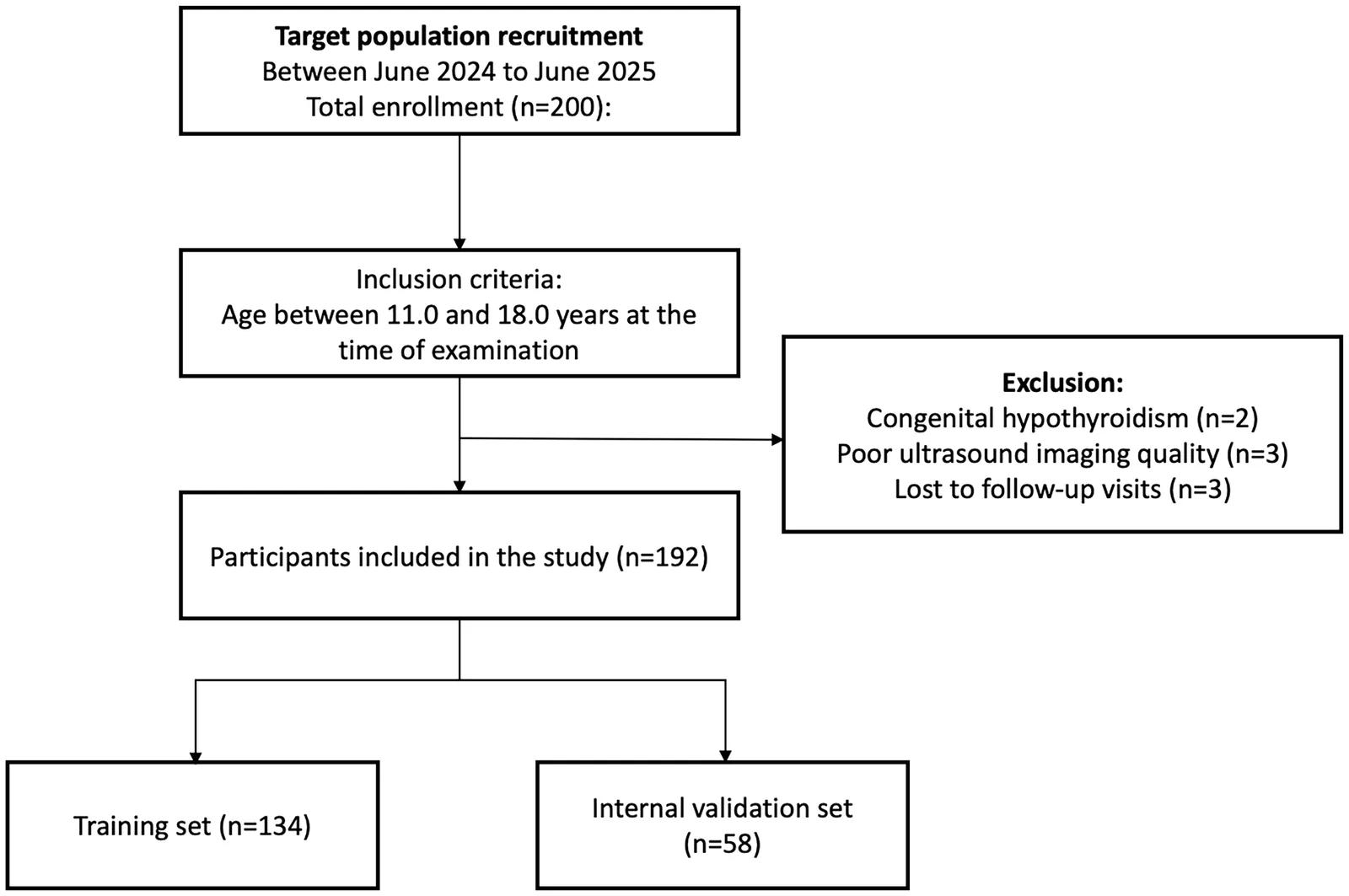
Flowchart of the inclusion and exclusion.

### Follow-up assessment and clinical characteristics

All participants underwent a one-year follow-up visit. Baseline clinical information was collected at study initiation including anthropometric measurements and laboratory examinations. Final height at the end of follow-up was also recorded. Attainment of Near-adult height (NAH) was defined as a height gain of less than 2 cm during the one-year follow-up period (12, 13). This cutoff reflects markedly reduced epiphyseal proliferative activity and serves as a practical operational surrogate for approaching the completion of linear growth in adolescents. Distinct from final adult height (FAH) (14), which typically requires complete epiphyseal closure or an annual height velocity < 1cm/year, this NAH definition indicates minimal residual growth potential. Considering the CA range of our cohort, and the fact that individuals in early-to-mid-puberty are unlikely to satisfy the FAH criterion, the participants were grouped according to whether they had reached NAH.

Height and weight were measured in the morning by a single physician using a standardized anthropometer in the Department of Endocrinology. Height was measured three times, and the mean value was calculated, provided the discrepancy was less than 0.2 cm. Body mass index (BMI) was calculated using the following formula: BMI (kg/m²) = weight (kg)/height² (m). To account for CA and sex variations, height was standardized by converting it to standard deviation scores (SDS) based on China’s national survey data (15, 16). BA was assessed using the Greulich-Pyle (GP) atlas. Laboratory tests were performed following established protocols at 8:00 a.m. All laboratory tests were performed together for each individual.

### Ultrasound examination and image acquisition

The standardized ultrasonographic examination was performed by three sonographers, each with more than 3 years of experience in musculoskeletal ultrasonography. Before participant enrollment, all sonographers completed protocol training to ensure consistency across examinations. The training emphasized standardized patient positioning, reproducible probe orientation relative to anatomical landmarks, avoidance of excessive probe pressure, and acquisition of a fixed view. During the examination, the participant was positioned naturally with the dorsum of the hand facing upward. Using the L6–24 boot-shaped probe on the GE Logiq E20 ultrasound system, the sonographer scanned the coronal plane of the ulnar styloid process to clearly visualize the metaphysis, secondary ossification center, and epiphyseal cartilage. To ensure acquisition consistency, a subset of examinations was periodically reviewed by a senior operator, who provided feedback on image-plane alignment and completeness of the required views. Any images that did not meet quality criteria were reacquired during the same visit whenever possible.

### Region-of-interest segmentation

All ultrasound images were exported from the GE LOGIQ E20 system in DICOM format, converted to NIfTI format, and normalized for grayscale intensity. The processed images were then imported into 3D Slicer (version 5.2.0) for manual ROI delineation. The ROI was defined to encompass the ulnar growth plate region, including the secondary ossification center, physis, epiphyseal cartilage, and the adjacent metaphyseal region. To standardize the metaphyseal extent of the ROI, the diaphyseal-side boundary was placed 5 mm from the metaphyseal margin of the physis toward the diaphysis. On the epiphyseal side, the ROI boundary was traced along the outer hyperechoic contour of the secondary ossification center and was completed by a tangent line drawn at its articular-facing end. The superficial boundary of the ROI was manually traced along the interface between the hyperechoic subcutaneous tissue and the underlying hypoechoic cartilage layer. The deep boundary was defined as the lowest clearly visible osseous/cartilaginous contour within the growth plate region on the ultrasound image. Thus, the final ROI encompassed the secondary ossification center, physis, epiphyseal cartilage, and a standardized 5-mm metaphyseal region (**Figure 2**).

**Figure 2:**
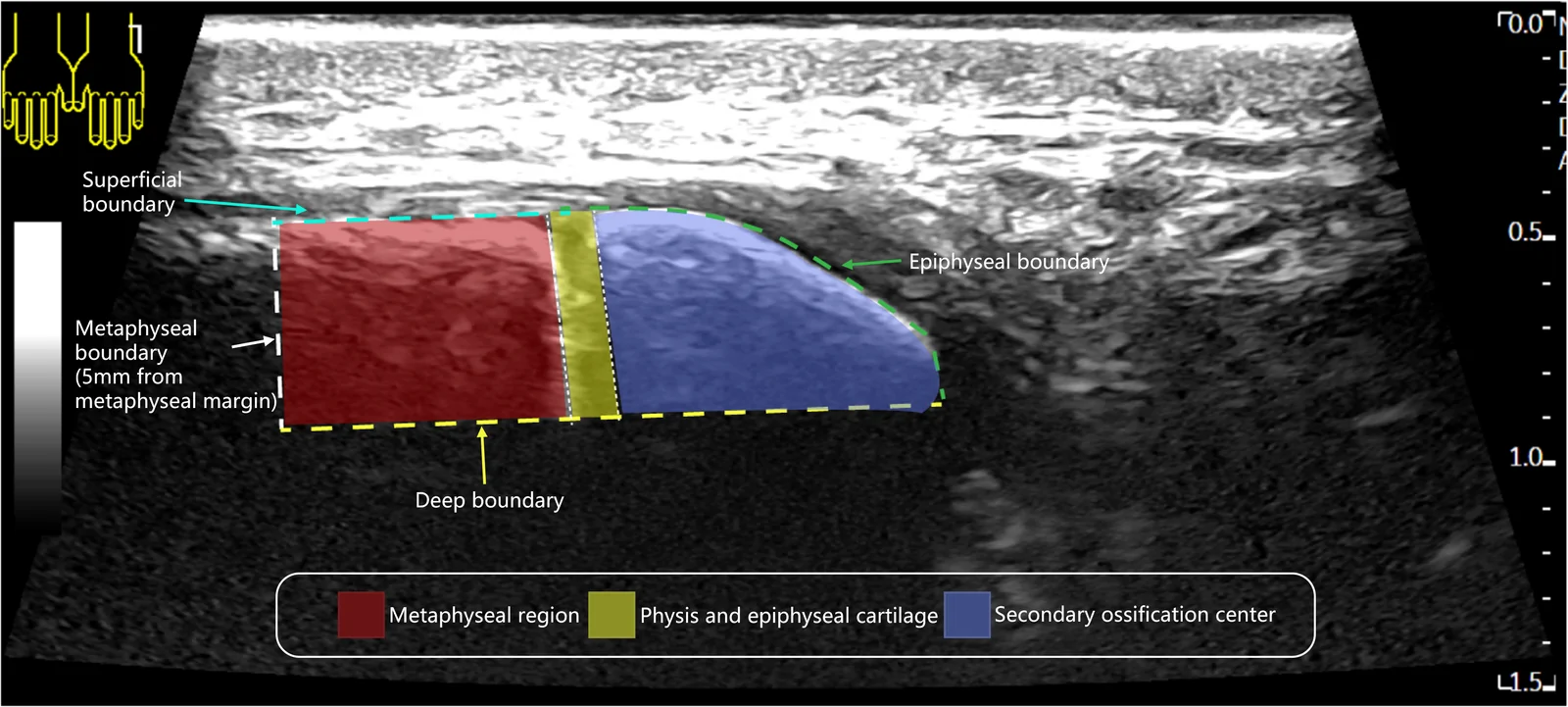
Manual delineation of the region of interest (ROI) on ultrasound. The ROI comprised the secondary ossification center (blue), physis and epiphyseal cartilage (yellow), and a standardized 5-mm metaphyseal region (red). The diaphyseal-side boundary was positioned 5 mm from the metaphyseal margin of the physis toward the diaphysis. The epiphyseal boundary followed the outer hyperechoic contour of the secondary ossification center and was completed by a tangent at its articular-facing end. The superficial and deep boundaries were defined by the interface between hyperechoic subcutaneous tissue and hypoechoic cartilage, and by the lowest clearly visible osseous/cartilaginous contour, respectively.

### Radiomic feature selection

Two researchers (Z.Z. and S.Y.) independently performed ROI delineation. Discrepancies in ROI boundaries between the two observers were resolved through consensus discussion. A third senior radiologist was consulted to reach a final decision if consensus could not be achieved. The consensus ROIs after resolving all disagreements were adopted for subsequent radiomic feature extraction. Radiomic feature extraction was conducted using PyRadiomics (version 3.1.0). Interobserver repeatability was evaluated via intraclass correlation coefficients (ICCs). Features with good reliability (ICC ≥ 0.75) were retained for further analysis. To reduce feature redundancy, pairwise Spearman correlation coefficients were computed among radiomic features. Features within each cluster exhibiting high collinearity (|ρ| > 0.9) were further pruned guided by random forest–derived feature importance, retaining the most informative feature within each correlated group. Feature selection was performed using a nested least absolute shrinkage and selection operator (LASSO) logistic regression framework. A nested cross-validation strategy was adopted, with a 5-fold outer loop for performance evaluation and a repeated 5-fold inner loop for hyperparameter tuning. The optimal regularization parameter was selected based on minimum cross-validated error in the inner loop. Within each outer fold, LASSO was refitted using the optimal parameter, and features with non-zero coefficients were retained to construct fold-specific radiomic signatures. Model performance was evaluated in the outer test sets using the radiomic score (Rad-score) and quantified by the area under the receiver operating characteristic curve (AUC). Feature robustness was assessed by selection frequency across outer folds. Features selected in ≥80% of folds were defined as stable features and used to construct the final model on the full dataset. The Rad-score for each participant was computed via the following formula: Rad-score=Σ (feature value × feature weight) + b_0_ (intercept).

### Model learning model development and evaluation

Seven machine learning algorithms, including support vector machine (SVM), stochastic gradient descent (SGD), k-nearest neighbors (KNN), random forest (RF), XGBoost, LightGBM, and logistic regression (LR), were implemented using the scikit-learn library (version 1.5.1). Stratified five-fold cross-validation with shuffling (random state = 42) was performed on the training set for hyperparameter optimization. Hyperparameters were selected according to the mean cross-validated area under the AUC. The predefined search space for each algorithm is provided in **Supplementary Table 1**. Variables identified as significant in the univariate analysis, together with the Rad-score, were used as input features for all machine learning models. For scale-sensitive algorithms, feature standardization was incorporated into the modeling pipeline using mean-centering and scaling by the standard deviation. Among the evaluated algorithms, LR was finally adopted given its competitive predictive performance and superior quantitative interpretability. Unlike black-box machine learning algorithms with opaque decision mechanisms, the LR model assigns explicit, fixed coefficients to each radiomic feature, clearly quantifying the direction and magnitude of each feature’s contribution to NAH prediction. Rad-score was standardized using mean-centering and scaling by standard deviation prior to model construction. Based on the types of input features, three predictive models were developed: a clinical model, a radiomics model, and a combined model. Model calibration was evaluated using calibration curves and the Brier score, whereas clinical utility was assessed by decision curve analysis (DCA). To improve model interpretability and facilitate clinical application, a nomogram and a forest plot were constructed to visualize the contribution of individual variables in the combined model. The overall technical workflow of the study is illustrated in **Figure 3**, including patient enrollment, image processing, radiomics feature extraction, feature selection, model development, and model evaluation.

**Figure 3:**
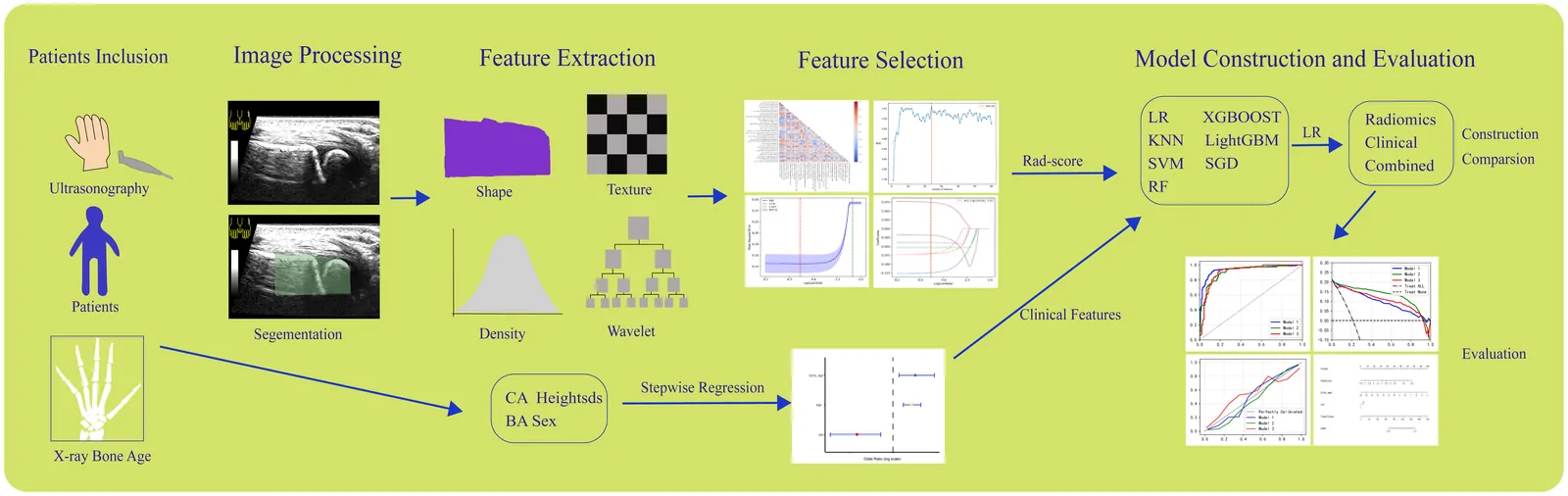
The technical workflow.

### Statistical analysis

For clinical characteristics, statistical differences were also assessed using normality testing, t-tests, and Mann–Whitney U tests. Continuous variables are presented as means ± standard deviations (SDs) for normally distributed data or as medians with interquartile ranges (IQRs) for non-normally distributed data. Categorical variables are presented as numbers (percentages). The clinical model, radiomics model and combined model were developed accordingly. The performance of all models, including the AUC, accuracy (ACC), sensitivity (SEN), specificity (SPE), positive predictive value (PPV), and negative predictive value (NPV), was evaluated. Differences in AUC values were compared using the DeLong test. A P value <0.05 was considered statistically significant. All analyses were performed using R (version 4.3.2) and Python (version 3.8).

## Result

### Baseline characteristics

A total of 192 participants were enrolled in this study and randomly split into a training set and an internal validation set at a 7:3 allocation ratio. The training set comprised 134 subjects, among whom 61 (45.5%) attained near-adult height (NAH), while the validation set included 58 participants, with 30 (51.7%) achieving NAH. Baseline demographic, anthropometric and biochemical characteristics were compared between the two cohorts, and no statistically significant intergroup differences were observed for all assessed variables (all P > 0.05), as summarized in **Table 1**.

**Table 1:** Comparison of baseline characteristics between the training set and validation set.

Variable	Training set (n=134)	Validation set (n=58)	P
Male [n (%) ]	82 (61.2)	31 (53.4)	0.400
School A/B (A%)	62/72 (46.3)	25/33 (43.1)	0.805
CA (years) [IQR]	13 [12, 13]	13 [12, 13]	0.199
BA (years) [IQR]	15 [14, 17]	15 [14, 17]	0.899
Height SDS	0.71 (1.38)	0.65 (1.18)	0.773
NAH + (%)	61 (45.5)	30 (51.7)	0.527
BMI SDS [IQR]	0.73 [0.09, 2.05]	0.57 [-0.07, 1.73]	0.398
TSH (μIU/mL) [IQR]	2.57 [1.90, 3.26]	2.60 [1.87, 3.60]	0.806
FT4 (pmol/L)	17.69 ± 2.41	18.07 ± 2.36	0.311
Vitamin D (ng/mL) [IQR]	15.70 [13.03, 20.38]	16.59 [14.61, 20.55]	0.174
Insulin (μIU/mL) [IQR]	13.15 [9.82, 18.98]	11.90 [7.40, 18.32]	0.148

In the training set, univariate analyses were performed to compare demographic, anthropometric, growth-related biomarkers between adolescents with non-attainment of NAH (NAH−, n = 73) and those with attainment of NAH (NAH+, n = 61), as summarized in **Table 2**. Significant intergroup disparities were detected in sex, CA, BA, and height SDS (all P < 0.001). No statistically significant intergroup differences were observed for BMI SDS, vitamin D, and insulin (all P > 0.05). TSH (P = 0.058) and FT4 (P = 0.096) only presented marginal group differences and did not reach the standard threshold of statistical significance. All variables with P < 0.05 in univariate comparisons were subsequently enrolled into multivariate binary logistic regression for stepwise variable screening.

**Table 2:** Univariate comparisons of demographic, anthropometric and growth-related biomarkers by NAH status in the training set.

	NAH- (n=73)	NAH+ (n=61)	P
Male [n (%) ]	58 (79.5)	24 (39.3)	<0.001*
CA (years) [IQR]	13 [12, 13]	13 [13, 14]	<0.001*
BA (years) [IQR]	14 [13, 15]	17 [15.5, 17]	<0.001*
Height SDS	0.26 ± 1.32	1.25 ± 1.25	<0.001*
BMI SDS [IQR]	0.76 [-0.21, 1.99]	0.66 [0.18, 2.12]	0.403
TSH (μIU/mL) [IQR]	2.72 [2.09, 3.23]	2.20 [1.82, 3.28]	0.058
FT4 (pmol/L)	17.37 ± 2.20	18.07 ± 2.60	0.096
Vitamin D (ng/mL) [IQR]	15.89 [13.17, 20.14]	15.54 [12.30, 20.50]	0.671
Insulin (μIU/mL) [IQR]	12.90 [9.29,17.80]	13.30 [10.00, 20.90]	0.768

Multivariate binary logistic regression was used to identify independent predictors of attaining NAH. Variables with P < 0.05 in the univariate analyses were first entered into the full model, including male sex, CA, BA, and Height SDS. Bidirectional stepwise selection based on the minimum Akaike Information Criterion (AIC) was then applied to optimize the model. In the first screening step, removing CA produced the lowest AIC value (AIC = 91.94), and CA was therefore excluded. The intermediate model retained male sex, BA, and Height SDS. Further analysis showed that Height SDS was not independently associated with NAH after adjustment for male sex and BA (OR = 1.49, 95% CI: 0.90–2.53, P = 0.130) (**Table 3**). The final optimal model retained only two significant independent predictors: male sex and BA. After mutual adjustment, male sex was significantly negatively associated with the odds of attaining NAH (OR = 0.054, 95% CI: 0.013–0.179, P < 0.001), indicating that males had a substantially lower probability of attaining NAH than females. BA was a strong positive independent predictor; each 1-year increase in BA was associated with a 3.34-fold higher likelihood of attaining NAH (OR = 3.34, 95% CI: 2.25–5.41, P < 0.001). Collectively, female sex and advanced BA independently increased the probability of attaining NAH, and these two clinical predictors were thus incorporated into the clinical prediction model.

**Table 3:** Multivariate binary logistic regression analysis of independent factors associated with NAH.

Variable	β	OR	95% CI lower	95% CI upper	P
Intercept	-17.266	-	-	-	-
Male	-2.92	0.054	0.013	0.179	<0.001*
BA	1.205	3.34	2.25	5.41	<0.001*
Height SDS	0.396	1.49	0.90	2.53	0.130

### Feature selection and construction of the Rad-score

A total of 475 robust radiomic features with intraclass correlation coefficients (ICC) ≥ 0.75 were extracted for downstream analysis. Nested LASSO regression was then applied to reduce feature dimensionality. **Figure 4A** shows the importance ranking of all candidate radiomic features, and three features with a selection frequency of at least 80% were ultimately retained: wavelet-LH_glcm_Imc2, wavelet-LL_ngtdm_Busyness, and wavelet-HL_firstorder_Energy. The optimal regularization parameter log(λ) was determined using the minimum mean squared error (MSE) criterion, as illustrated by the MSE curve in **Figure 4B**. The coefficient trajectories of all radiomic features across different log(λ) values are presented in **Figure 4C**, where features crossing the vertical red dashed line retained non-zero regression coefficients and were included in the construction of the Rad-score. The Rad-score formula derived from the three selected features was defined as follows: Rad-score = -0.1283 × wavelet-LH_glcm_Imc2 − 0.1976 × wavelet-LL_ngtdm_Busyness − 0.0548 × wavelet-HL_firstorder_Energy + 0.4552. Subsequent intergroup comparison revealed a statistically significant difference in Rad-score between the NAH− and NAH+ groups (P < 0.001), as shown in the box plot in **Figure 4D**.

**Figure 4:**
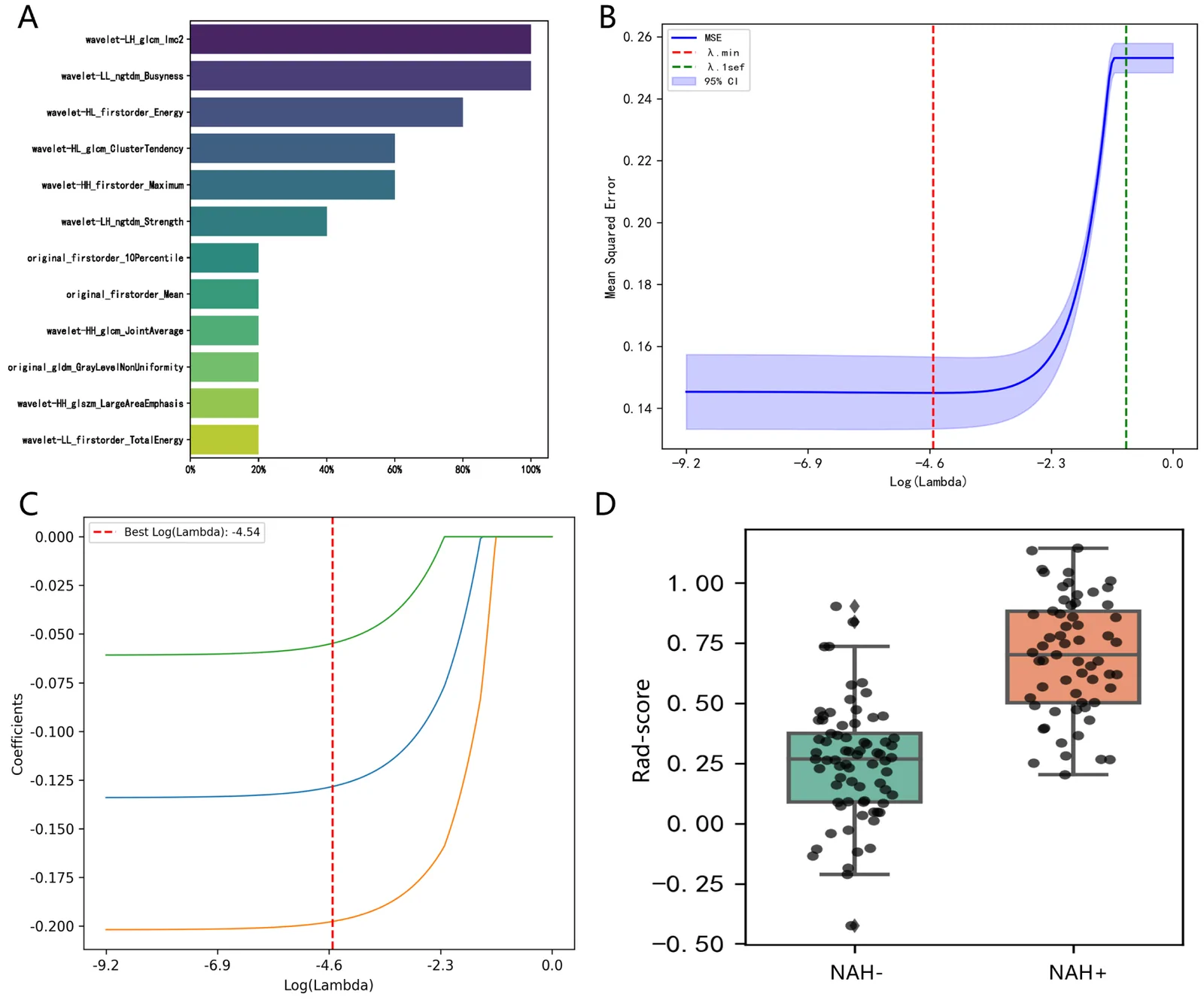
Radiomic feature screening and Rad-score comparison via nested LASSO regression. **(A)** Bar plot showing the relative importance of candidate radiomic features; darker bar shades correspond to higher feature contribution weights. **(B)** Mean squared error (MSE) curve for tuning parameter log(λ). The red dashed line indicates the optimal log(λ) with minimal MSE, and the green dashed line denotes λ.1se. **(C)** Coefficient trajectory curves of radiomic features across varying log(λ) values. The vertical red dashed line marks the optimal λ for feature selection. **(D)** Box plot of Rad-scores between NAH− and NAH+ groups in the training set.

### Machine learning model performance comparison

We incorporated the Rad-score, sex, and BA to construct predictive models for NAH attainment. Seven machine learning algorithms were applied, and their discriminatory performance was evaluated using receiver operating characteristic (ROC) curves (**Figure 5**). In the training cohort, all algorithms demonstrated excellent discriminative ability, with AUC values ranging from 0.942 to 1.000. In the validation set, these models maintained strong generalizability, with AUC values ranging from 0.906 to 0.980. Among all candidates, LR, SVM, and SGD showed consistently high and stable predictive performance in both the training and validation cohorts.

**Figure 5:**
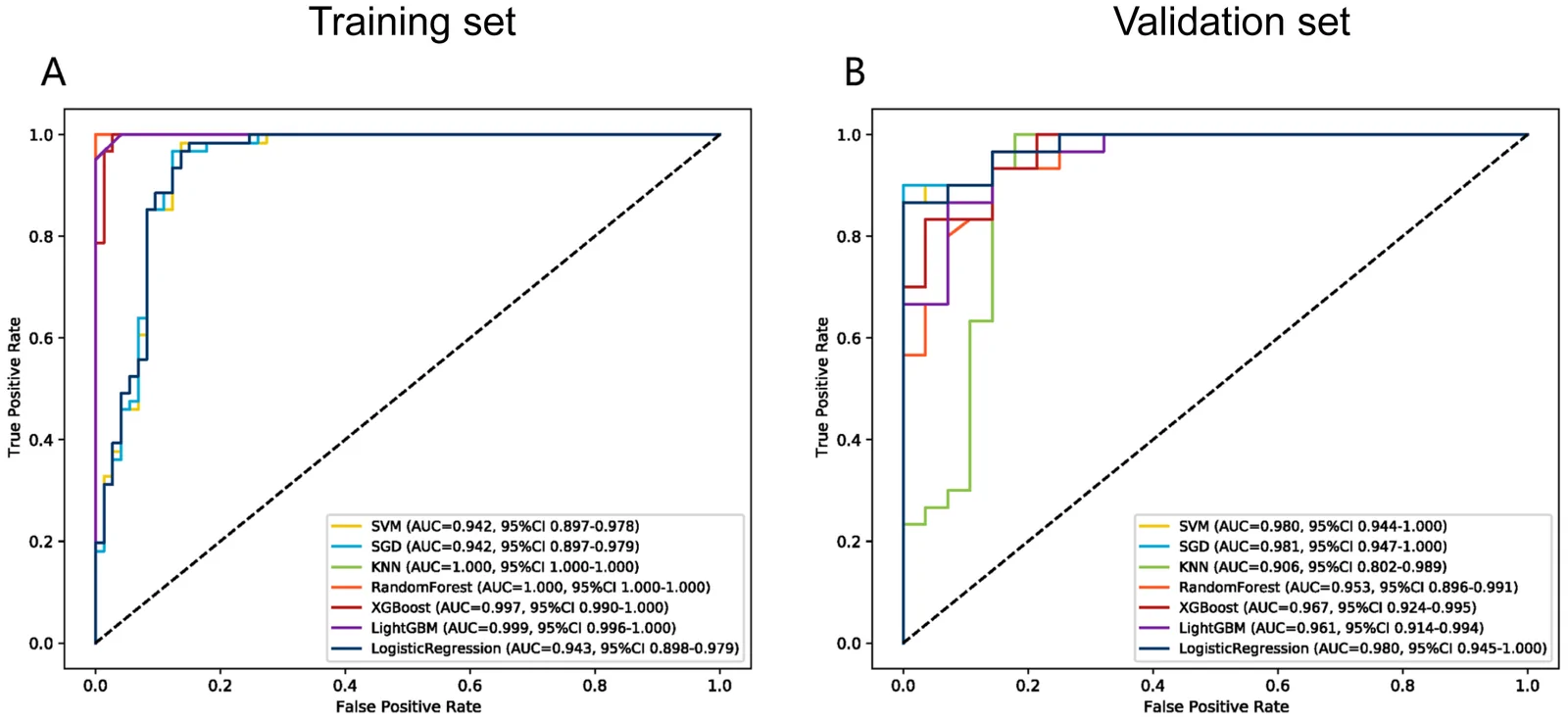
ROC curves comparing the discriminative performance of seven machine learning algorithms. **(A)** ROC curves of all models in the training set. **(B)** ROC curves of all models in the validation set.

### Performance of clinical, radiomics and combined prediction models

The LR model was selected as the optimal algorithm for subsequent model evaluation. Based on clinical and radiomic features, we developed three predictive models: a clinical model incorporating sex and BA, a radiomics model constructed using the Rad-score, and a combined model integrating sex, BA, and the Rad-score. The performance of all models in the training and validation cohorts is shown in **Table 4**. All models achieved excellent discriminative ability with mean AUC values > 0.90 in both cohorts, with no significant performance degradation in the validation set. The combined model outperformed the single models, achieving the highest AUC values (training: 0.943, 95% CI: 0.900-0.979; validation: 0.981, 95% CI: 0.948-1.000) and the lowest Brier scores in both cohorts.

**Table 4:** Predictive performance of three models in the training and validation cohorts.

Cohort	Model	AUC (95% CI)	SEN (%)	SPE (%)	ACC (%)	PPV (%)	NPV (%)	Brier
Training set	clinical	0.925 (0.873-0.965)	93.4	87.7	90.3	86.4	94.1	0.096
	radiomics	0.901 (0.844-0.949)	82	89	85.8	86.2	85.5	0.125
	combined	0.943 (0.9-0.979)	96.7	87.7	91.8	86.8	97	0.086
Validation set	clinical	0.954 (0.896-0.994)	100	85.7	93.1	88.2	100	0.082
	radiomics	0.940 (0.868-0.992)	90	92.9	91.4	93.1	89.7	0.108
	combined	0.981 (0.948-1)	90	100	94.8	100	90.3	0.069

Calibration curve and decision curve analyses further confirmed the superior predictive accuracy and clinical utility of the combined model. Calibration curves were used to evaluate the agreement between the predicted probabilities and the observed outcomes (**Figures 6A, B**). In both the training and validation cohorts, the combined model showed the closest alignment with the ideal reference line, indicating the best calibration performance. Decision curve analysis was then performed to assess the net clinical benefit of each model across a range of threshold probabilities (**Figures 6C, D**). The combined model demonstrated superior predictive value for attaining NAH compared with the clinical and radiomics models, consistently providing greater net clinical benefit across a broad range of threshold probabilities, particularly around 0.75.

**Figure 6:**
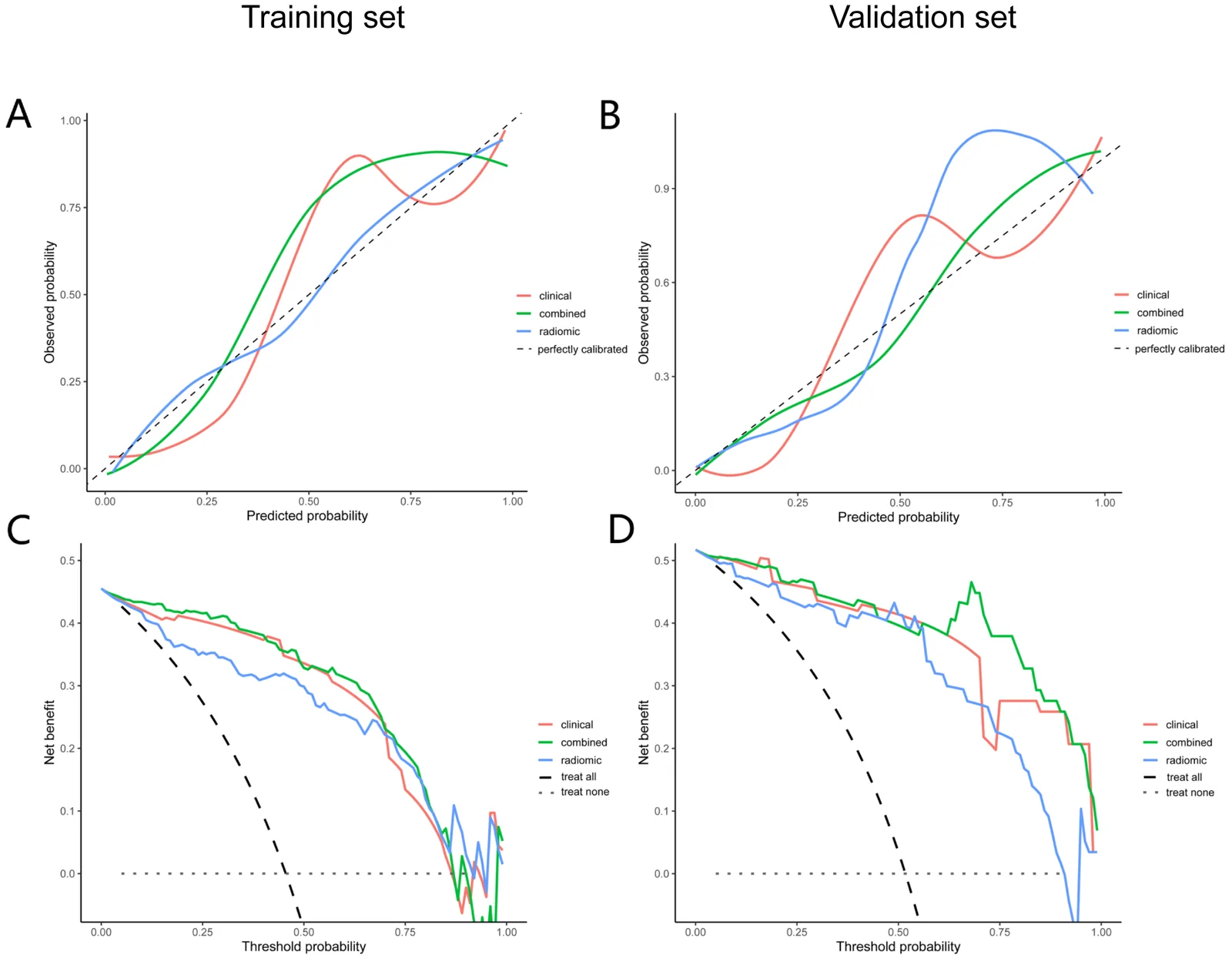
Calibration and decision curve analysis for the clinical, radiomics, and combined predictive models. **(A, B)** Calibration curves of the three models in the training set and validation set. **(C, D)** Decision curves illustrating the net predictive benefit across varying threshold probabilities in the training set and validation set.

### Model interpretation

Multivariable logistic regression of the combined model identified the contribution of three independent predictors. Per standard deviation (SD) elevation in Rad-score conferred the strongest positive association with NAH+ status (OR = 3.28, 95% CI: 1.37–9.03, P = 0.013), followed by BA, where each 1-year increment raised the odds of attaining NAH (OR = 2.65, 95% CI: 1.73–4.40, P < 0.001). Male sex was a significant negative predictor relative to female sex (OR = 0.15, 95% CI: 0.036–0.52, P = 0.005). These multivariate effect sizes and confidence intervals are displayed in the forest plot (**Figure 7**).

**Figure 7:**
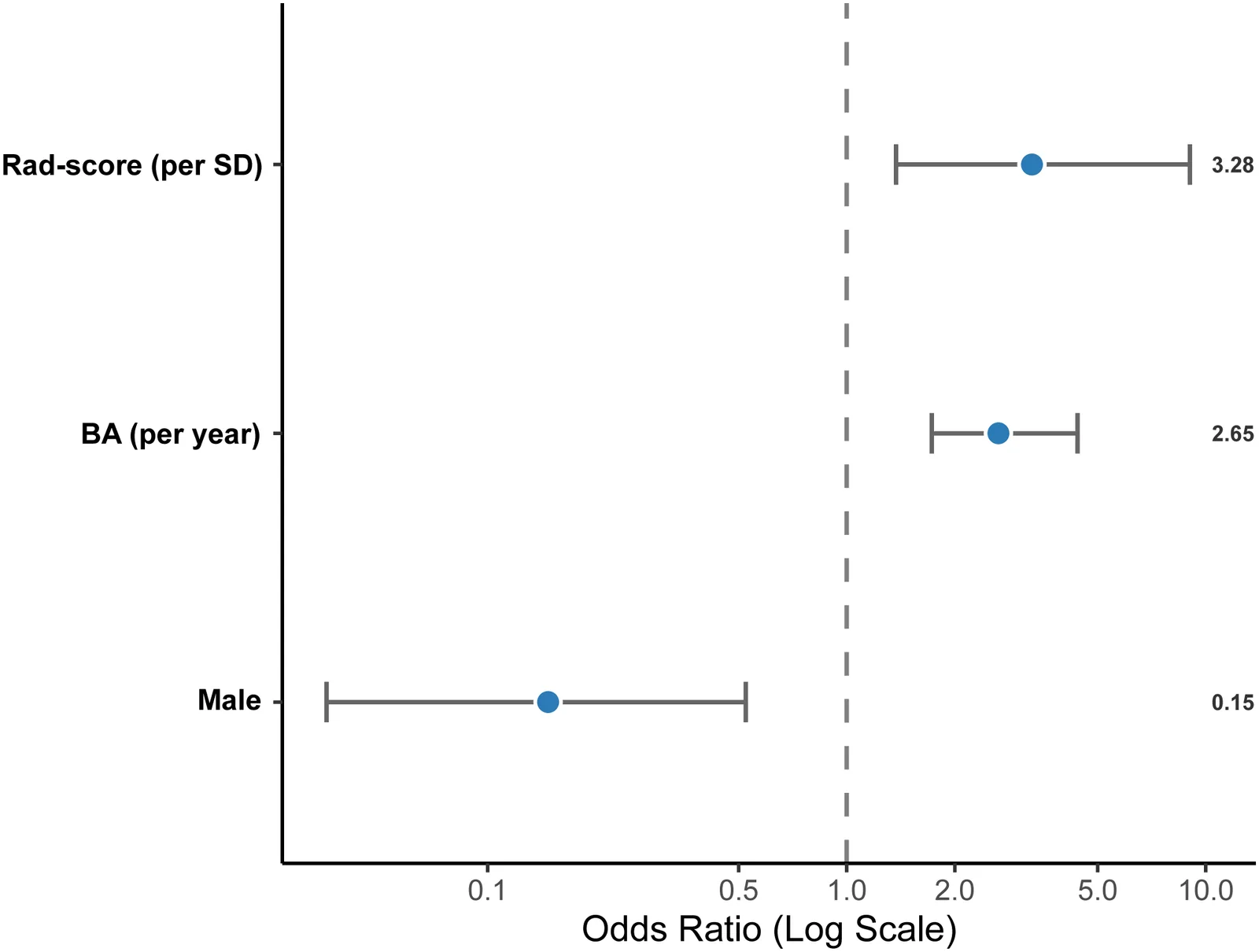
Forest plot displaying adjusted odds ratios of independent predictors in the combined logistic regression model for attaining NAH.

Using the regression coefficients from the combined model, we constructed a nomogram to facilitate individualized quantitative risk prediction of attaining NAH (**Figure 8**).

**Figure 8:**
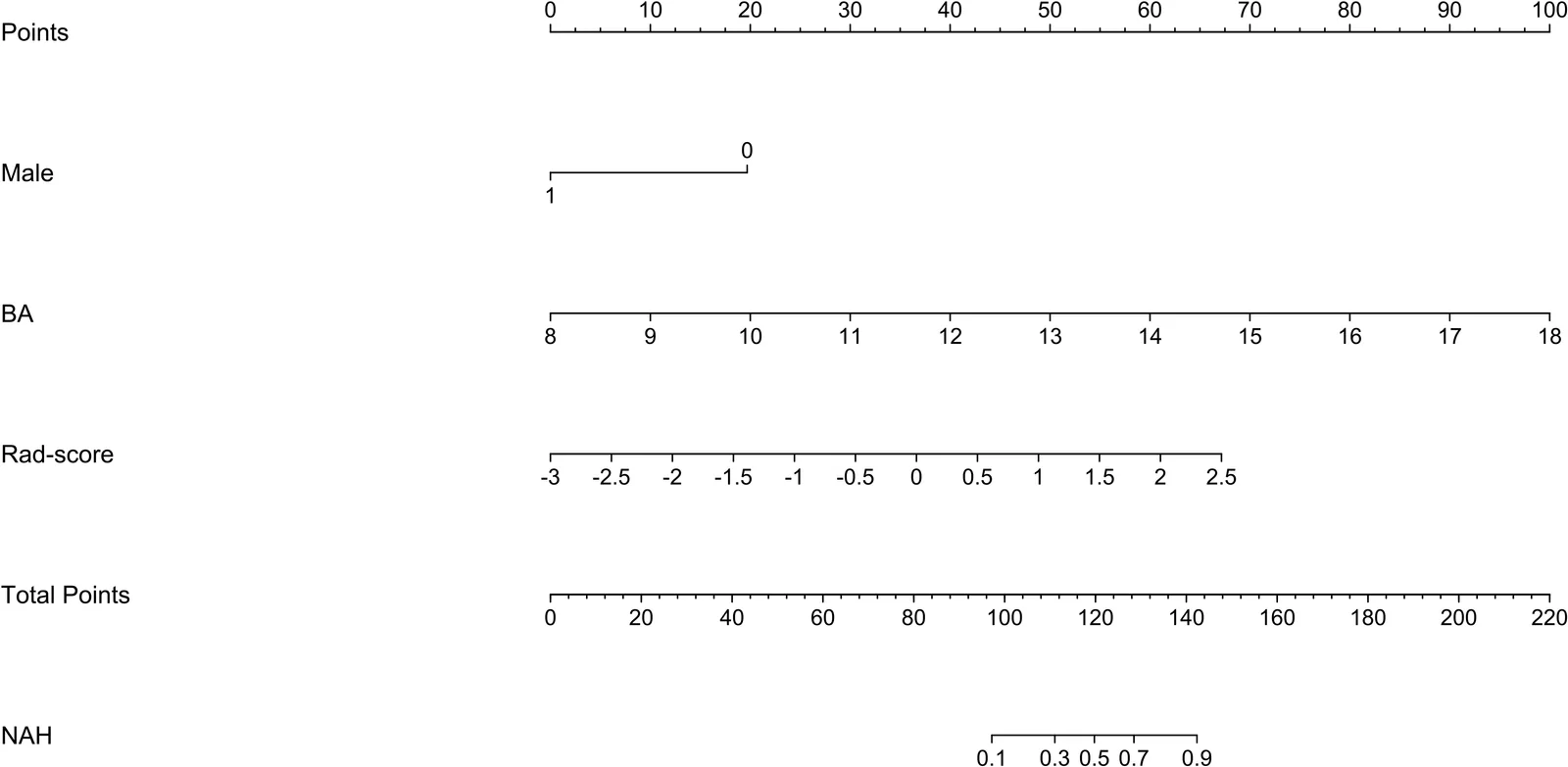
Nomogram of the combined model in the training set.

## Discussion

This study introduced and validated a novel method based on ultrasound radiomics for predicting the attainment of NAH in adolescents. The core finding was that a radiomic signature (Rad-score), derived from texture and morphological features of ultrasound images of the ulnar physis, demonstrated significant predictive performance for growth potential (AUC = 0.940, 95% CI: 0.868-0.992). The Rad-score, combined with key clinical parameters, achieved superior predictive accuracy compared to models using clinical data or radiomic features alone. Specifically, the combined model exhibited high AUC [0.981 (0.948-1.000), 90% and 100%] in classifying adolescents as likely to attain or not attain a predefined NAH threshold, thus offering a promising tool for individualized growth assessment.

The rationale for using ultrasound to assess growth potential is well-founded, as it provides a non-ionizing alternative to traditional radiographic BA assessment (7). Previous ultrasound-based methods have primarily focused on scoring ossification centers or measuring growth plate thickness, which are indirect proxies for skeletal maturity (7). In contrast, our approach extracts a comprehensive, quantitative radiomic signature that captures subtle tissue characteristics not discernible by human eyes or simple measurements. The three stable radiomic features retained in the final model reflect progressive biological changes during ulnar growth plate maturation. First, wavelet-LH_glcm_Imc2 characterizes image textural heterogeneity, which may correspond to microstructural disorganization of hypertrophic chondrocyte layers during growth plate senescence. Second, wavelet-LL_ngtdm_Busyness quantifies local grayscale intensity variation, which may reflect increased extracellular collagen deposition and gradual matrix calcification during ossification progression. Third, wavelet-HL_firstorder_Energy represents overall ultrasound signal energy distribution, which is consistent with decreased cartilage water content and reduced cellular density as epiphyseal cartilage matures and ossifies. These texture-based biomarkers provide non-invasive quantitative reflections of histological and physiological changes in adolescent skeletal maturation. This aligns with the broader trend in medical imaging toward radiomics and AI-driven analysis, which has shown promise in various fields, including BA estimation (17). The superior performance of our radiomics model compared to simpler ultrasound metrics reported in the prior research (18) underscores the potential of extracting high-dimensional data from conventional ultrasound images.

Furthermore, our study complements and extends existing research that uses other biomarkers to predict growth. Anthropometric models -for example, the target height prediction method proposed by Zeevi et al. (19) -largely depend on genetic potential inferred from parental height and population averages. In contrast, our ultrasound radiomics approach directly evaluates the current biological status and residual growth capacity of an individual’s growth plate region. Studies on conditions such as idiopathic scoliosis have examined a variety of approaches, including anthropometric models (20), simplified radiographic staging systems such as the Thumb Ossification Composite Index (TOCI) (21), and biochemical biomarkers such as Collagen X (CXM) (22). Notably, Welborn et al. identified CXM as a direct measure of endochondral ossification and a strong predictor of height velocity (22). Our radiomics-based method, which directly targets growth plate region, may capture complementary information related to the same underlying biological process of endochondral ossification (23). While CXM reflects a dynamic systemic marker, our approach provides a localized structural assessment. This distinction highlights a multifaceted view of growth potential, in which no single method is likely to be universally superior.

The strengths of this study are multifaceted. First, ultrasound avoids the ionizing radiation associated with conventional X-ray-based BA assessment, making it particularly suitable for repeated long-term monitoring in pediatric and adolescent populations. Second, we developed and compared three predictive models by integrating ultrasound radiomics with clinical variables using multiple machine learning algorithms. Statistical analysis confirmed that the Rad-score remained an independent predictor even after adjustment for clinical factors, demonstrating that radiomics adds prognostic value beyond traditional BA indicators. Third, because our model was built on complete longitudinal follow-up data, it can reliably predict adolescents’ overall growth trajectory and directly target the clinically meaningful endpoint of near-adult height (NAH), which better reflects real-world needs for evaluating growth potential. Unlike static BA assessment, which captures only current skeletal maturity, this integrated approach offers greater practical value for long-term growth prognosis. Finally, we derived a visual, interpretable nomogram from the optimal combined model, transforming complex radiomic and machine learning outputs into a simple scoring tool that supports rapid individualized risk stratification in routine pediatric practice.

However, several limitations of this study should be acknowledged. First, the Rad-score was derived solely from the distal ulnar growth plate and may not fully reflect systemic skeletal maturation, given the variability in maturation across long bones and trunk bones. Participants were enrolled from an eastern Chinese city; regional growth differences relative to the GP atlas may partly explain the observed advancement in BA. In fact, reported discrepancies between GP-atlas-derived BA and CA across different regions (24) are one of the reasons we sought new methods to assess adolescent growth and development. Second, the model predicted a binary NAH endpoint rather than continuous growth-velocity trajectories for dynamic monitoring, and classification based on one-year height gain is subject to confounding by sex and pubertal maturation. Finally, the model was developed and internally validated using data from two geographically proximate hospitals, which limits its generalizability across broader populations. In addition, the relatively small sample size and the complexity of machine learning-based modeling raise concerns about potential overfitting, so the high internal validation AUC may be somewhat optimistic. Therefore, multicenter external validation in larger, multiethnic populations is essential before application.

## Conclusion

In conclusion, this study constructed and validated a novel ultrasound radiomics machine learning model to predict the attainment of NAH among adolescents. Through standardized image processing, rigorous feature screening, and multimodal model comparison, we verified that the combined model integrating Rad-score and BA achieved optimal predictive efficacy. Radiation-free ultrasound supports repeated dynamic monitoring of pediatric growth potential, and the derived interpretable nomogram further facilitates convenient individualized risk stratification in routine clinical practice. Although restricted by single-center recruitment and insufficient external validation, this non-ionizing quantitative framework provides an objective supplementary tool for evaluating adolescent growth trajectory, and lays a foundation for subsequent multi-cohort verification and multi-dimensional data fusion research in pediatric growth disorder precision management.

## Data Availability

The raw data supporting the conclusions of this article will be made available by the authors, without undue reservation.
