# Supplementary material for: A novel method based on ultrasound radiomics for predicting attainment of near-adult height in adolescents
Source: Front Endocrinol (Lausanne). 2026 Sep 18;17:1925196. doi: 10.3389/fendo.2026.1925196 (PMC13630604; doi:10.3389/fendo.2026.1925196)
Supplement: Supplementary file 1 [file Table1.docx]

Supplementary Table 1. Hyperparameter search spaces for the seven machine learning algorithms

| **Algorithm** | **Hyperparameter** | **Candidate values** |
| --- | --- | --- |
| SVM | C | 0.1, 1, 10, 100 |
|  | Kernel | linear, rbf |
|  | Gamma | scale, auto |
| SGD | Loss | log_loss |
|  | Penalty | l1, l2 |
|  | Alpha | 0.0001, 0.001, 0.01 |
| KNN | Number of neighbors | 1–10 |
|  | Weights | uniform, distance |
| RF | Number of estimators | 10, 50, 100, 200 |
|  | Maximum depth | 3, 5, 10, 20 |
| XGBoost | Number of estimators | 10, 50, 100 |
|  | Maximum depth | 3, 5, 10 |
|  | Learning rate | 0.01, 0.1, 0.2 |
| LightGBM | Number of estimators | 10, 50, 100 |
|  | Maximum depth | 3, 5, 10 |
|  | Learning rate | 0.01, 0.1, 0.2 |
| LR | C | 0.1, 1, 10 |
|  | Penalty | l1, l2 |
|  | Solver | liblinear (fixed) |
